# Development of an algorithm to guide management of cardiorespiratory arrest in a diving bell

**DOI:** 10.1016/j.resplu.2024.100724

**Published:** 2024-07-18

**Authors:** Graham Johnson, Andrew Tabner, Nicholas Tilbury, Alistair Wesson, Gareth D. Hughes, Rebecca Elder, Philip Bryson

**Affiliations:** a University Hospitals of Derby and Burton NHS Foundation Trust, Royal Derby Hospital, Uttoxeter Road, Derby DE22 3NE, UK; b University of Nottingham School of Medicine, Queen’s Medical Centre, Nottingham NG7 2UH, UK; c Independent; d North West School of Anaesthesia, Manchester, UK; e TAC Healthcare, Wellheads Crescent, Aberdeen AB21 7GA, UK

**Keywords:** Resuscitation, Algorithm, Austere environment, Mechanical CPR, Pre-hospital, Diving bell

## Abstract

**Aim:**

The management of cardiorespiratory arrest in a diving bell presents multiple clinical, technical, and environmental considerations that standard resuscitation algorithms do not address, and no situation-specific algorithm exists. The development and testing of an algorithm to guide the management of cardiorespiratory arrest in a bell is described.

**Methods:**

An iterative approach to algorithm development was used. Phase 1 involved a small multidisciplinary group and took place in a simulation centre and a decommissioned diving bell. The algorithm was then refined in a purpose-build simulation complex with repeated simulation by a group of divers, and with input from industry experts. ALS principles were followed unless contextual or technical factors necessitated deviation.

**Results:**

Clinical and technical aspects of the resuscitation are addressed. Key priorities that conflict with standard ALS principles are: prioritisation of rescue breaths; use of mechanical CPR when available; and the provision of CPR with the casualty in a seated position where necessary.

**Conclusion:**

This is the first algorithm to guide the delivery of resuscitation in a diving bell. It incorporates adapted ALS principles and available data concerning compression technique effectiveness, and was informed by industry and clinical expertise. It provides guiding principles that can be adapted to setting-specific needs, and we would encourage its industry-wide international adoption.

## Highlights

• This is the first algorithm guiding the provision of resuscitation in a diving bell.
• Setting-specific considerations required adaptation of existing approaches.
• A key change from ALS is the prioritisation of rescue breaths via iGel.
• Mechanical CPR is preferred, but manual chest compression techniques are validated.
• Algorithm development involved divers, cross-industry expertise and stakeholders.

## Introduction

**Fig. 1:**
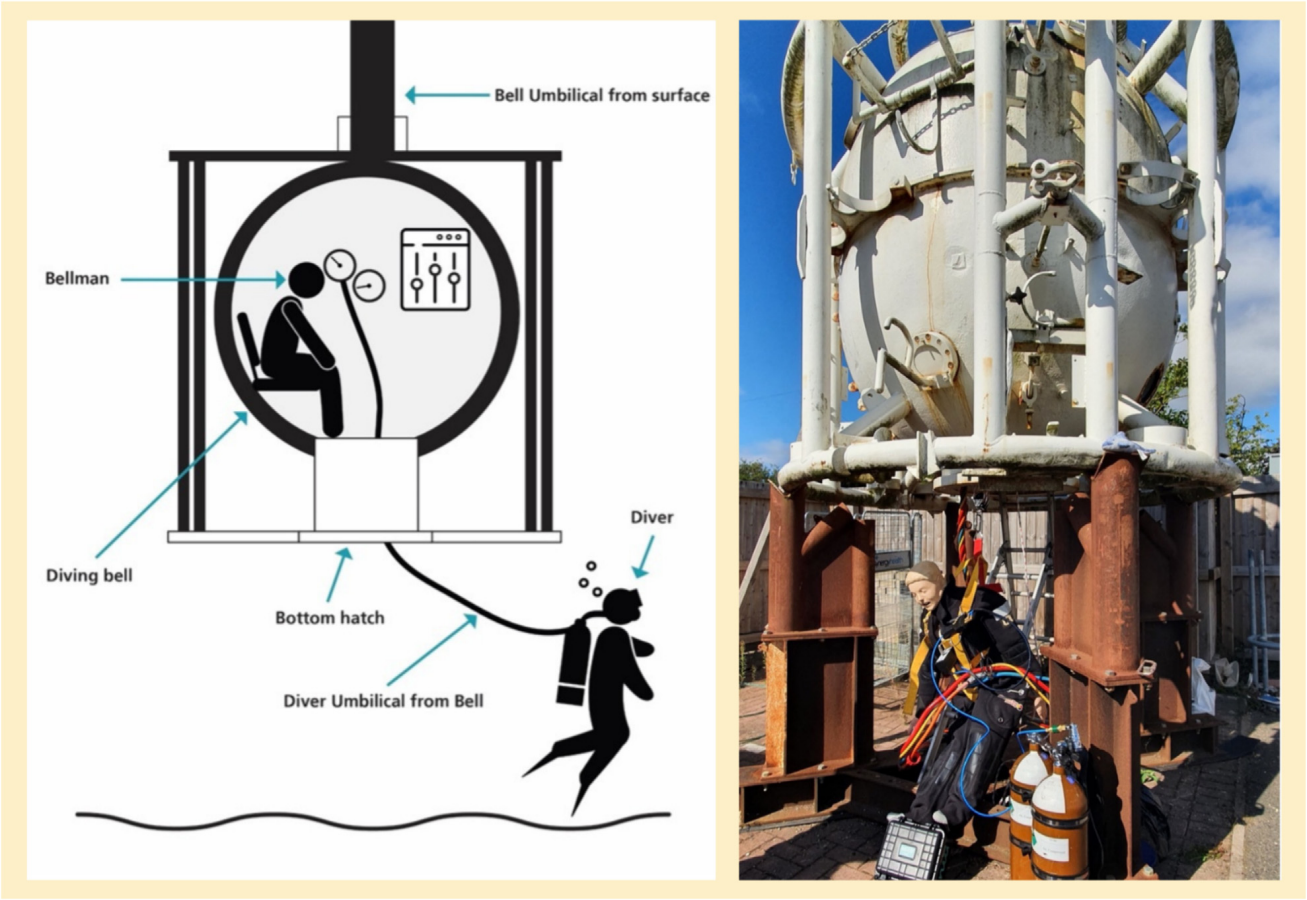
a (left). Diving bell schematic. A diving bell in cross-section. b (right). Diving bell exterior. The diving bell on site at the Royal Derby Hospital, with a Resusci Anne QCPR suspended below, together with some equipment.

Commercial saturation diving requires divers to live in a hyperbaric environment for up to a month. They spend their time in a saturation (sat) chamber on board a ship, and are transported to and from their work site, 20–300 m below the surface, in a diving bell (Fig. 1). Exiting the hyperbaric environment requires a decompression period lasting many days, depending on depth.^1^

An ageing diving workforce^2^ with an increasing prevalence of (sometimes undisclosed^3^ chronic illness means that sudden cardiorespiratory arrest is an increasing concern,^4^ with notable recent incidents^5^, ^6^; internationally, a mean of 11 deaths occur during commercial diving activities each year (range 2–26, time period 2015–2022).^7^, ^8^ Sat chambers are comparatively spacious, contain multiple rescuers, may have access to a defibrillator, and allow immediate access to medical advice; arrests can therefore be managed using conventional protocols.

In contrast, diving bells are cramped (internal diameter < 3 m) and contain 2–4 divers and extensive equipment. There may be no flat surface on which to lay a casualty, and there is currently no suitable defibrillator available. If arrest occurs whilst the casualty is working outside of the bell then casualty recovery and equipment removal poses additional challenge. Extrication times from work site to ship can be up to 40 min, delaying access to further help.

No situation-specific algorithm to guide the management of cardiorespiratory arrest in a diving bell exists. There are published techniques for manual chest compression delivery in this environment (seated knee-to-chest^9^ and upright CPR [submitted]); they allow chest compression delivery comparable to conventional CPR by manikin assessment, although their clinical effectiveness is not evidenced. There is also a mechanical CPR (mCPR) device, the NUI NCCD,^10^ designed for the saturation diving setting; it delivers compressions comparable to conventional CPR and other mCPR devices.^11^

This manuscript describes the development and refinement of an algorithm to guide the management of cardiorespiratory arrest occurring in a diving bell setting.

## Methods

Algorithm development and refinement took place in two phases:

1. **Development phase**

**Setting:** The emergency department and simulation centre of the Royal Derby Hospital, together with a decommissioned diving bell (equipped with cameras for simulation review) was installed on site (Fig. 1).

**Study team:** Emergency medicine doctors/researchers and nurses (and life support course instructors), a diving and hyperbaric medicine physician, an offshore medic, and a saturation diver/dive medical technician (DMT).

**Equipment:** Chest compression and ventilation efficacy were assessed using a Resusci Anne QCPR intelligent manikin, outfitted with relevant diving equipment as practicable.

## Algorithm development

An iterative approach to algorithm creation included: round table discussion; design; testing with direct observation; video debrief; and revision and refinement. This process continued until team members were satisfied that no further refinements were indicated.

2. **Refinement phase**

**Fig. 2:**
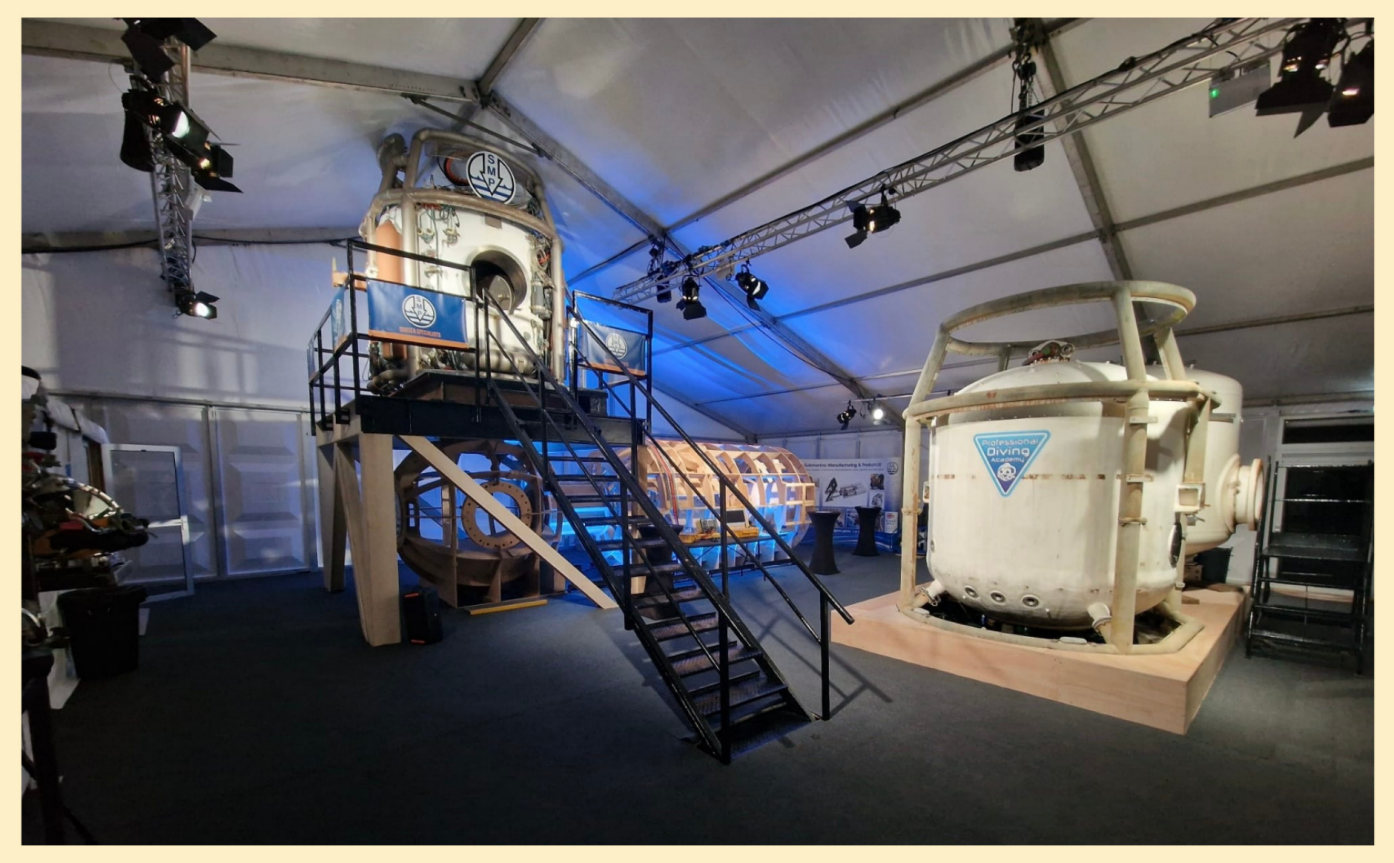
Purpose-built simulation complex, Dunoon. Facilities included both bottom-mating (left) and side-mating (right) diving bells, simulated TUP (“wet pot”) and saturation chamber.

**Setting:** A purpose-built simulation complex at the Professional Diving Academy, Dunoon, comprising two diving bells (bottom- and side-opening) with representative chambers (Fig. 2).

**Study team:** As above, plus industry representatives from multiple organisations (including the International Marine Contractors’ Association (IMCA)), 11 commercial saturation divers and 2 dive supervisors.

**Equipment:** As above, plus a Ruth Lee manikin (weighted for realistic casualty handling simulation), and representative diving equipment.

The refinement phase involved a 3-day programme with continuous, iterative algorithm amendment:

**Day 1:** Baseline assessment of diver knowledge and practice, key skill training (e.g. knee-to-chest CPR, NCCD application), algorithm familiarisation and initial refinement.
**Day 2:** Iterative simulation of algorithm sections (e.g. airway management and rescue breaths).
**Day 3:** Iterative full algorithm simulations (including live simulated casualties). Assessment of manual compression technique efficacy in the diving bells.

## Results

**Fig. 3:**
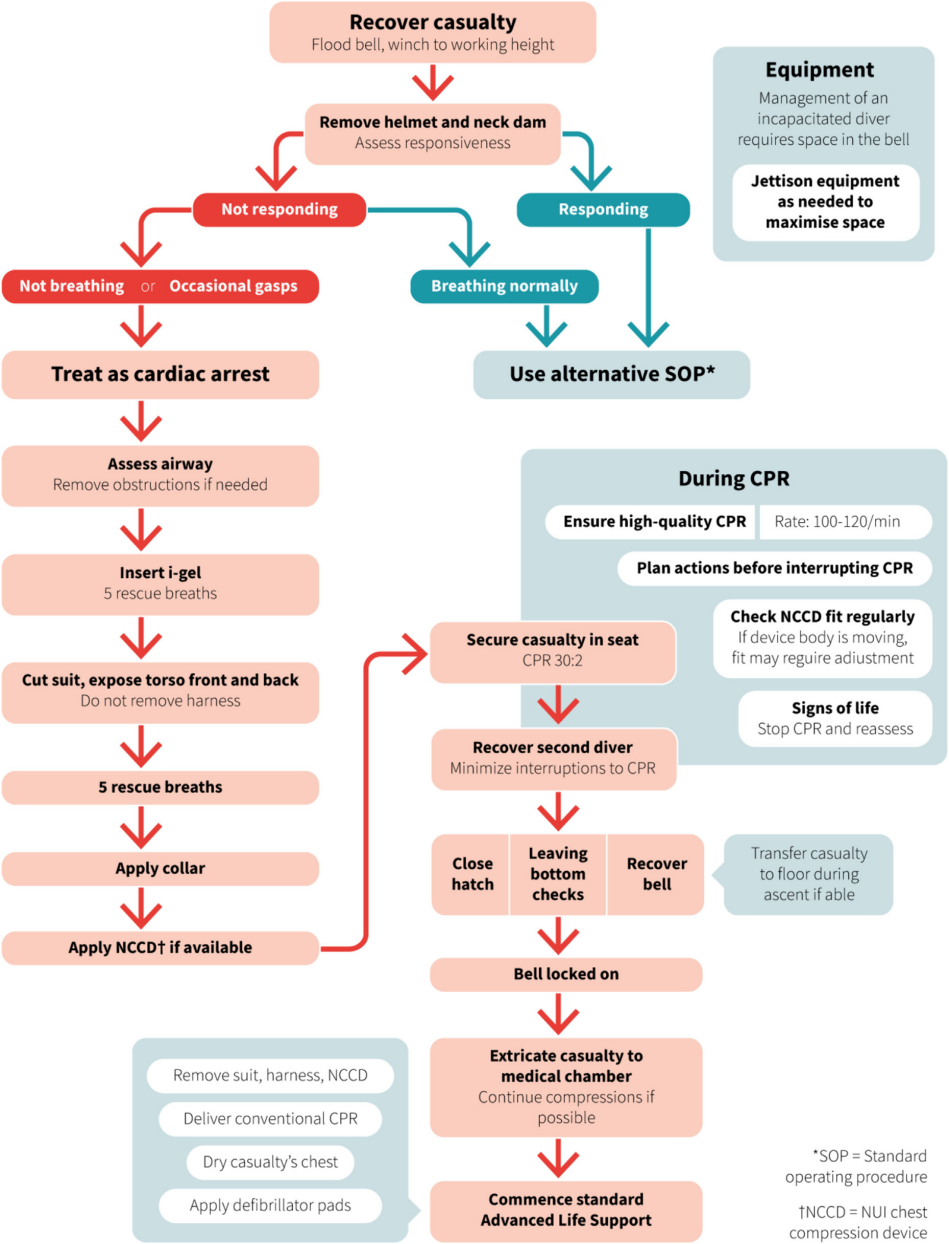
Algorithm for the resuscitation of a diver in cardiorespiratory arrest in a diving bell.

The algorithm can be seen in Fig. 3; recommended actions, including rationale where these deviate from ALS protocols, are detailed below. It is impractical to account for all permutations of bell design, equipment availability, and diver/casualty location at the time of incident, in a single algorithm. This algorithm should therefore be considered a high-level strategy providing a framework and key interventions to support the development of local Standard Operating Procedures (SOPs). It assumes that both the casualty and the second diver are outside of the bell (their most likely locations) at the time of incident; this represents the most logistically challenging situation for scenarios involving a single casualty.

### Algorithm actions

#### Recover casualty, flood bell, winch to working height

Industry SOPs for the recovery of an incapacitated diver are established (and will be modified in view of this algorithm); they include dialogue and support between rescuers in the bell and staff on board the support vessel,. The bellman (a diver who remains in the bell during a dive) and second diver collaborate to recover the casualty to underneath the bell and attach them to the bell’s winch. Extraneous equipment (e.g. bail-out system^12^ should be removed from the casualty and discarded.

The casualty should be winched through the bell’s trunking and bottom hatch to the bellman’s working height to enable casualty management, assisted by the second diver (where possible) who will by necessity remain outside of the bell at this point. Flooding the bell with water to the maximum safe height will help to facilitate this.

#### Remove helmet and neck dam, and assess casualty

The bellman removes the casualty’s helmet and neck dam (waterproof neck seal) and assesses their responsiveness and breathing; if both unresponsive and either not breathing or with agonal respirations then they are deemed to be in cardiorespiratory arrest. Signs of life at any point during management should prompt reassessment.

Pulse assessment is challenging even for trained providers,^13^ and is likely to be impossible in this setting. The risks of delaying resuscitation outweigh those of unnecessary treatment.

#### Assess airway

**Fig. 4:**
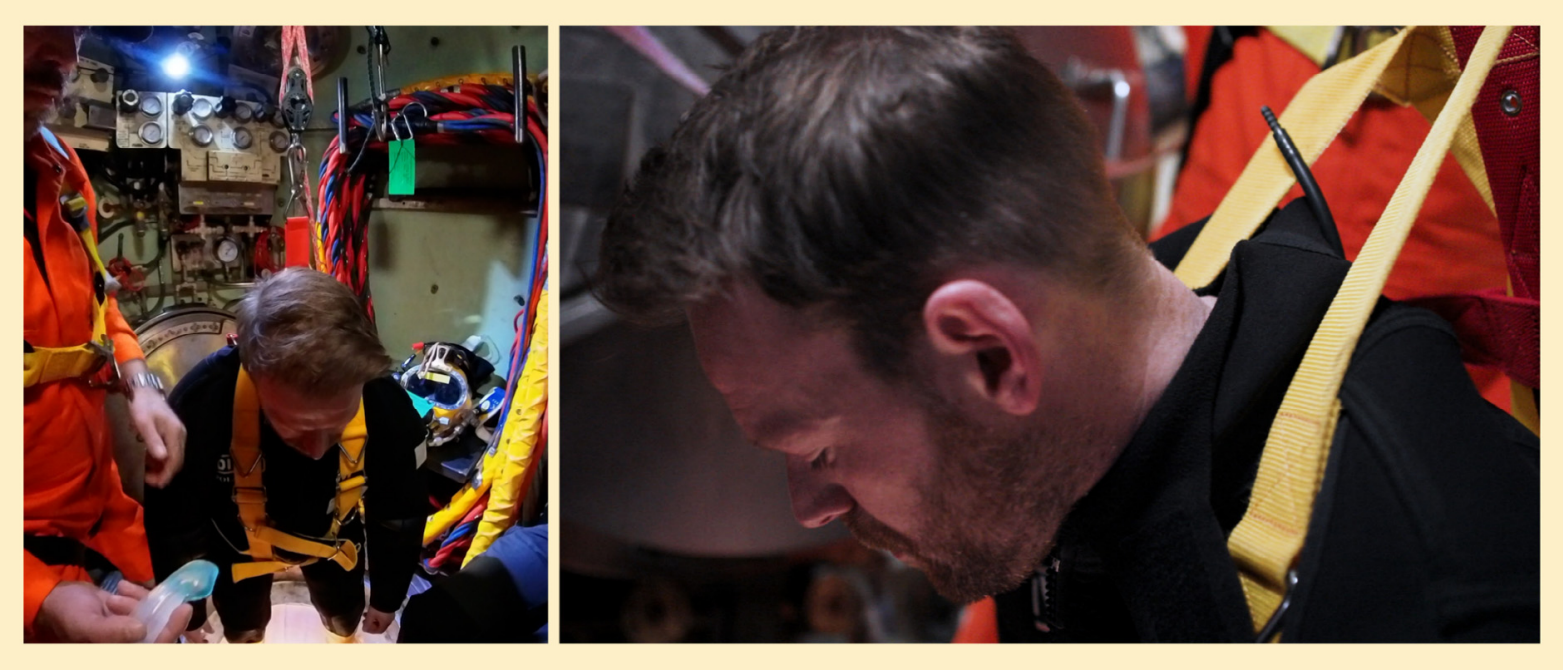
Casualty position when suspended from hoist.

Assessment for trauma, foreign body (e.g. loose dentures) and vomitus. Positional drainage will likely assist with clearance of vomitus given the hoisted position of the casualty with head slumped forwards (Fig. 4); suction can be employed if available.

#### Insert iGel, deliver 5 rescue breaths

An iGel should be inserted and 5 rescue breathes delivered whilst the casualty is suspended from the winch; rescue breaths are then delivered approximately every 60 s until effective CPR can be commenced.

Prioritisation of rescue breaths over early chest compressions represents a deviation from ALS protocol, implemented because hypoxia is a likely cause for arrest in this setting (e.g. gas supply interruption/contamination). Early ventilations may therefore result in return of spontaneous circulation^6^ and can be delivered rapidly, causing little delay to other resuscitative techniques, but could be significantly delayed by the steps required to initiate chest compressions.

Divers demonstrated that they could insert an iGel with appropriate technique with minimal instruction, and reliably achieved effective ventilations of the manikin during simulations.

An iGel was selected because:

• An oropharyngeal airway is likely to fall out given casualty position, and an iGel may help support airway patency despite neck flexion
• It facilitates repeated rescue breaths with minimal interruption to other activities
• It allows the use of a bag-valve-mask if available; this would be more challenging with other airway management approaches for the non-expert user

It is acknowledged that an imperfect seal may be achieved, or ventilations may be challenging to deliver; this was felt to be less likely than if mouth-to-mouth rescue breaths were employed (with or without the use of a pocket mask).

#### Cut suit, expose torso front and back, do not remove harness

A hot water suit reduces manual chest compression efficacy, and the NCCD cannot be reliably applied with the suit intact^11^; it should be removed by cutting down both arms from the neck opening and peeling down front and back sections. It was found to be significantly quicker to perform these actions whilst the casualty was suspended from the harness than once they were seated. The harness should be left intact to enable casualty extrication. Time suspended from the harness should be minimised to reduce risk of suspension syndrome^14^; external compression of the casualty's legs from water in the flooded bell will further reduce this risk, and the benefits of keeping the casualty suspended for these actions were felt to outweigh the risks.

#### Administer 5 rescue breaths

Suit removal was found to take approximately 1 min during repeated trials; rescue breaths were therefore included in the algorithm again as a further prompt, as they were frequently forgotten due to rescuer task fixation.

#### Apply collar

**Fig. 5:**
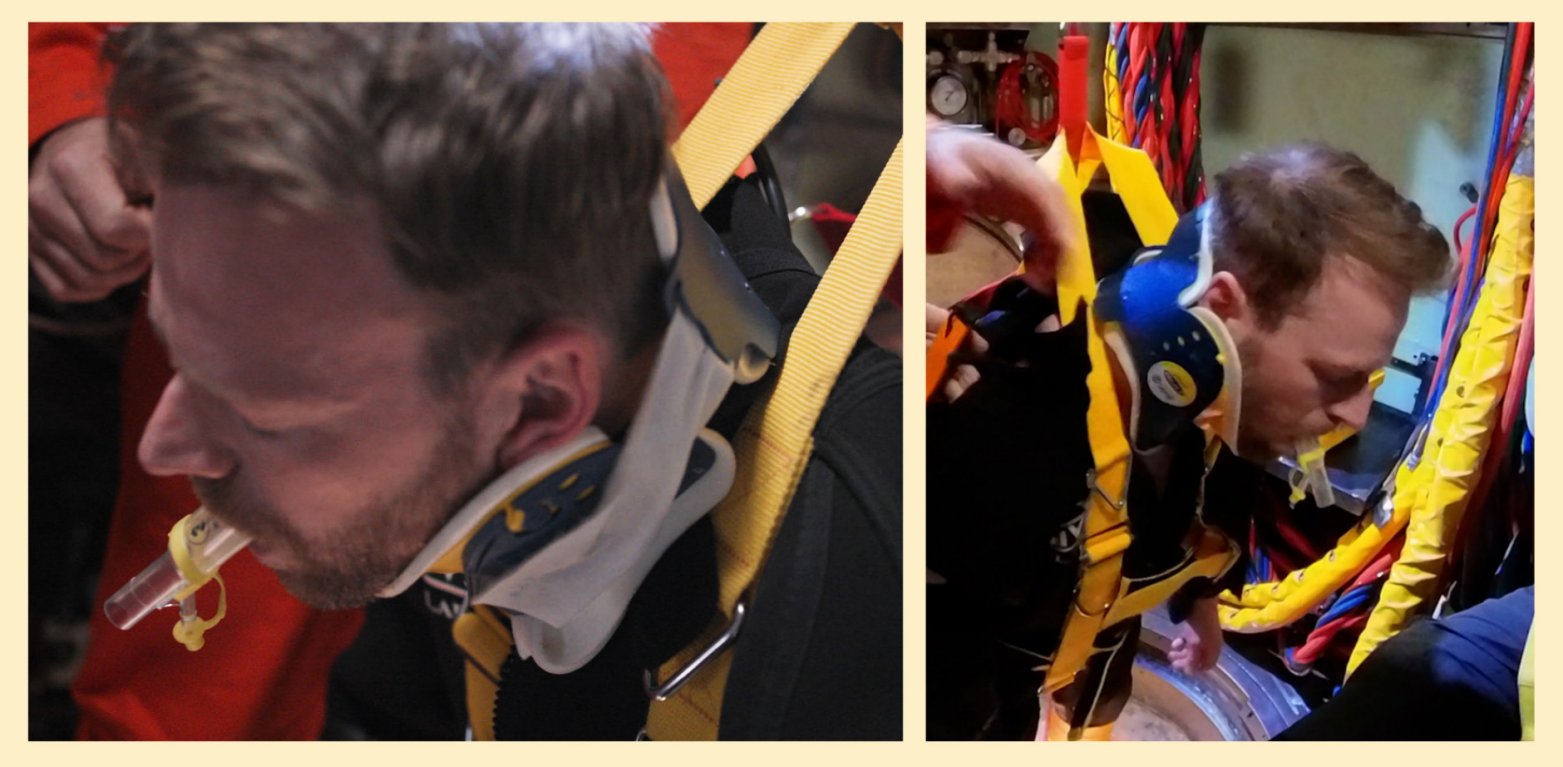
Casualty position once collar and iGel applied.

The collar is required for head stabilisation, rather than neck immobilisation; it was found challenging to manage the airway or deliver chest compressions without a collar applied. The rigid collar was superior to a soft collar in live casualty simulations, and divers were able to apply these effectively. The Laerdal Stifneck Select was used in the “No Neck” position and found to be suitable for this purpose for all divers present (Fig. 5). Collar application should follow iGel insertion to avoid restricting mouth opening.

#### Apply NCCD if available

In healthcare settings mCPR is no more effective than manual CPR, but it is recommended in situations where: provider resource is lacking; prolonged resuscitation is likely; or environmental constraints make manual CPR challenging.^15^ These circumstances are all met in a diving bell resuscitation, and use of mCPR is recommended whenever possible.

NCCD application whilst the casualty is suspended in the harness was found to be far quicker than once the casualty was in a seated position, and could be performed by a single operator in approximately 1 min. The NCCD strap can be placed either over or under the harness without interfering with either device efficacy or harness utility; this finding may be harness-dependent and should be assessed at an organisational level.

NCCD application was sometimes challenging in the bell, and it was felt that design alterations may enable more consistent placement; these recommendations were provided to the manufacturer and an updated device is expected.

#### Secure casualty in seat, administer CPR 30:2

The casualty should be secured in a seat with shoulders restrained against the bell wall to enable chest access for CPR; this functionality was noted to be variable between bells, but is required for effective CPR and must be addressed by the industry for effective resuscitation to be feasible.

It is not possible to lie the casualty on the floor of the bell until “leaving bottom” checks have been completed and the bottom hatch is closed; this can be a relatively prolonged process. CPR must therefore be commenced with the casualty in a seated position.

**Fig. 6:**
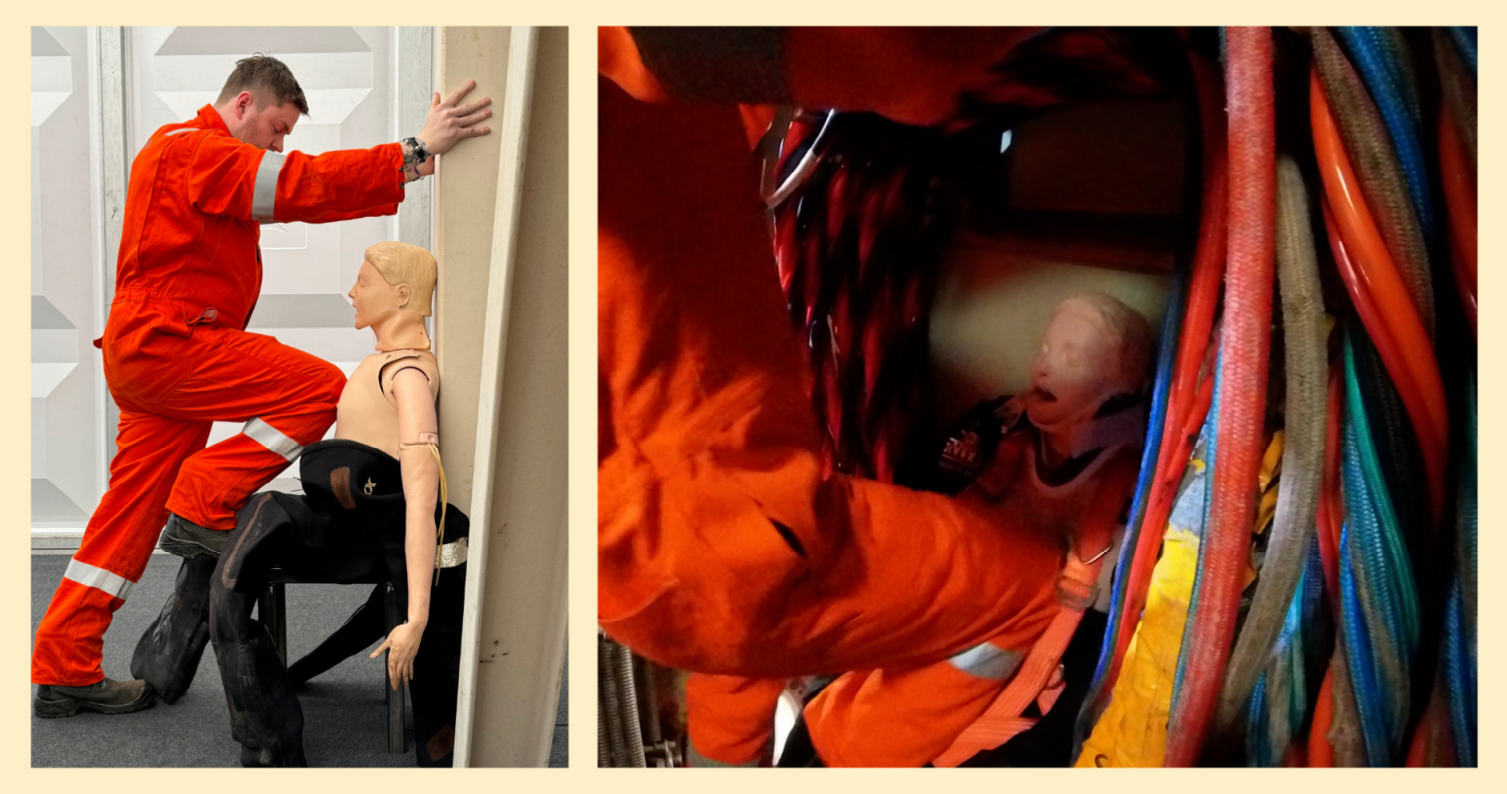
Knee-to-chest CPR simulated on land (left) and in a diving bell (right).

If no mCPR device is available then manual CPR must be delivered; the only published technique with supporting efficacy data is seated knee-to-chest CPR.^9^, ^16^ Diver ability to deliver knee-to-chest CPR in a diving bell (Fig. 6) was assessed during this study, with 7 diver pairs delivering seated knee-to-chest CPR to an intelligent manikin for 4 min, alternating as needed. They achieved a median depth of 55 mm (range 47–56), with good rate, recoil, and position; full data can be seen in Table 1. This supports the proposition that this technique is teachable, as this represents a significant improvement when compared to metrics achieved by novice providers in a previous publication.^9^

**Table 1:** Manikin efficacy metrics for seated knee-to-chest CPR delivered by divers in a diving bell.

**Pair**	**Depth (mm)**	**Depth (%)**	**Recoil (%)**	**Rate** **(/min)**	**Av. Pause (s)**
1	55	98	99	101	3
2	56	99	81	120	4
3	55	97	98	113	3
4	56	97	68	103	5
5	47	47	98	113	4
6	51	71	87	112	4
7	51	83	67	115	5
**Median**	**55**	**97**	**87**	**113**	**4**

**Fig. 7:**
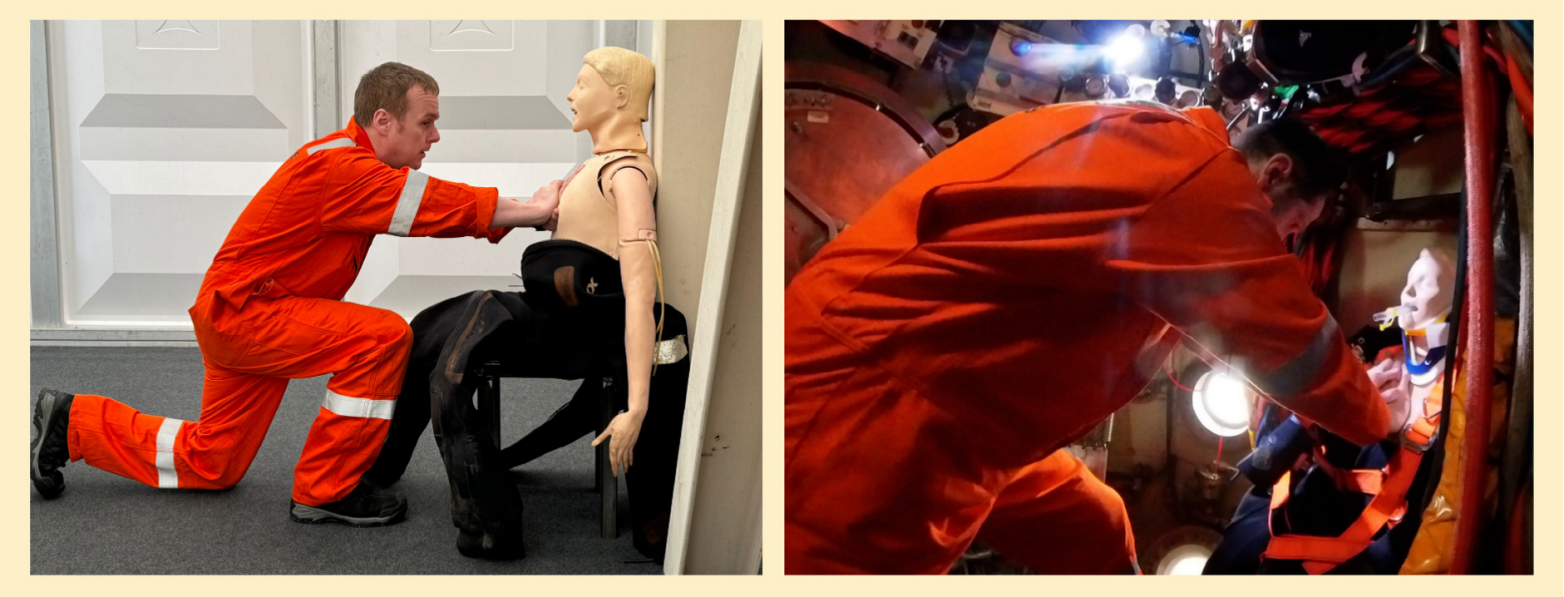
Upright (“Dunoon”) CPR, simulated on land (left) and in a diving bell (right).

Divers proposed a previously undescribed variation on conventional CPR (termed “upright CPR” or “the Dunoon method”), with the provider in a standing/high-kneeling position braced against the bell, delivering compressions using their hands (Fig. 7). Stance will be governed by the diver height/casualty position. This technique is the topic of letter to the editor^17^; it was found to be similarly effective to seated knee-to-chest CPR, and the divers reported it to be more sustainable.

Manual CPR can therefore be delivered using either the seated knee-to-chest or the upright method, with providers able to select a method based upon ergonomic considerations, and vary their choice as required during a resuscitation.

#### Recover second diver, minimise interruptions to CPR

The second diver, if outside the bell at the time of the incident, will have to re-enter the bell before it can begin its ascent to the ship. It will not be possible to deliver manual CPR whilst the diver enters through the bottom hatch, and the bellman will therefore pause CPR and assist the second diver to re-enter as quickly as possible. Conversely, compressions with the NCCD can be continued during this process; the second diver may still require assistance with their equipment.

Any equipment that is hampering rescue efforts should be discarded through the bottom hatch, maximising available space.

#### Close hatch, leaving bottom checks, recover bell

Once CPR is established, the priority is to return the casualty to the sat chamber; interruptions to CPR should be minimised. Continuous chest compressions are not recommended even with an iGel in situ, as assessment of air leak/ventilation efficacy by non-experts is challenging.

#### Casualty positioning during ascent

The casualty should be moved to a supine position if bell design permits; mCPR can be delivered on an irregular floor, but manual CPR (conventional or straddle^18^ requires a flat floor for safety and efficacy. The winch should be used to support repositioning.

Whilst some evidence for the effectiveness of (and even improved outcomes with) head-up CPR exists, it is not sufficiently evidenced in human studies to advocate keeping the casualty seated (particularly in the absence of a supine “priming” period) if the possibility of supine CPR exists.^19^

#### Casualty extrication

Some diving bells have a side hatch for exit to the sat chamber, and the casualty can remain on the floor whilst this hatch is opened to allow extrication. Others have only a bottom hatch, and the casualty must therefore be winched to their seat to enable the hatch to be opened. The NCCD was shown to be effective during winch transfers; long tubing for the NCCD driving gas must be considered during bell outfitting, and brief pauses in compressions during transfers through trunking are inevitable.

Interruptions to mCPR during extrication should be minimised; manual CPR cannot continue whilst transferring the casualty from the bell to the sat chamber. Once in the chamber, all equipment should be removed from the casualty’s torso; leaving the NCCD in situ was found to encourage poor defibrillator pad placement. The casualty’s chest should be dried, and thereafter management should follow ALS protocols; the NCCD can be re-applied if prolonged CPR is envisaged. The initial priority will be attaching a defibrillator and assessing the casualty’s rhythm to enable prompt defibrillation if required; the “lock to shock” time should be minimised, and the transfer process should be planned and drilled at a setting-specific level.

## Discussion

This is the first algorithm designed to guide the management of cardiorespiratory arrest in a diving bell. It has been developed using high-fidelity simulation in collaboration with key stakeholders, and has its foundations in ALS principles. It provides a framework from which companies can develop their own SOPs specific to their operating environments. Some deviations from ALS protocol have been required; these were influenced by the availability of expertise or equipment, the limitations of the environment, or context-specific medical considerations.

The availability of an mCPR device suited to the environment has significantly influenced the development of this algorithm. Whilst mCPR is not superior to manual CPR in a traditional healthcare setting,^15^ many context-specific factors render manual CPR in a diving bell significantly challenging to deliver. Compression provider fatigue in this setting is inevitable^20^; there are a limited number of compression providers, bell recovery times are long, and the incident may occur after a full day of manual labour. Casualty recovery from the dive site to the bell is also extremely strenuous. We therefore recommend mCPR as the compression technique of choice, and its provision, along with relevant training, should be the industry standard.

The prognosis of cardiorespiratory arrest in a diving bell is poor regardless of management; it is poor with the best possible medical care,^21^ and the saturation diving setting presents multiple challenges that reduce the likelihood of a positive outcome. Nevertheless, an evidence-based approach to management is vital, both to ensure the best chance of survival for the casualty in case of a reversible pathology (e.g. hypoxia, arrhythmia) and to minimise the psychological trauma caused to fellow divers who are forced to act as rescuers. It was acknowledged by all present during the refinement phase that at some point in a resuscitation you may be treating the rescuers as much as the casualty; nevertheless, continuation of resuscitation efforts throughout bell ascent, and until a full assessment of the casualty has taken place in the sat chamber, was unanimously felt to be in both the casualty and the rescuers’ best interests. Resuscitation cessation decisions can then be made in accordance with existing ALS guidance by staff with the knowledge and experience to support them.

The development of this algorithm is timely; divers in the North Sea range from 23-69 years old (mean 46), with a trend over time towards increasing mean age; all North Sea dive contractors currently have active divers > 65 years old.^2^ With increasing age comes an increasing likelihood of sudden cardiac arrest,^4^, ^22^ and the saturation diving industry has a responsibility to ensure its employees are afforded the best care possible in the circumstances.

## Limitations

It is likely impossible to assess the impact of this algorithm on outcomes of cardiorespiratory arrest in a diving bell; they are thankfully rare, and will be so heterogenous in aetiology and clinical detail as to make meaningful comparisons between cases impossible. However, the experiences of users who deliver CPR using this algorithm can be used to shape future versions.

The algorithm does not address multiple casualty scenarios. Simultaneous cardiorespiratory arrests in this setting are almost certain to be environmental in origin, caused by either trauma or hypoxia (e.g. interrupted gas supply). The management of traumatic cardiac arrest in this setting is almost certainly futile, and divers will not have the necessary skillset to perform trauma-related interventions in a diving bell. Furthermore, the delivery of high-quality chest compressions to multiple casualties by a single rescuer is physically impossible. We therefore suggest that the priority in this situation should be early rescue breaths and rapid extrication, but acknowledge that the logistical challenges are potentially overwhelming.

## Conclusions

This is the first algorithm developed to guide the resuscitation of a diver who suffers a cardiorespiratory arrest in a diving bell. It represents a step-change in the industry’s ability to provide them with the best possible medical care. It provides guiding principles that can be adapted to setting-specific needs, and we strongly encourage both its international adoption and the provision of appropriate training and equipment to enable its effective delivery.

## Funding statement

The project would not have been possible without generous support from the following organisations:

Professional Diving Academy, Submarine Manufacturing and Products, Boskalis, NUI, BP, DFS Diving, Equinor, IMCA, K-Subsea, KD Marine, Rever, RockSalt Subsea, Shelf Subsea, Subsea 7, TechnipFMC, Total Energies, Unique Hydra (PTY) Ltd, Well-Safe Solutions.

These funders had no role in the design or implementation of the protocol or the decision to publish.

International SOS and TAC Healthcare sponsored the work of one of the authors: Dr P Bryson.

## Approvals

This study was sponsored by the University Hospitals of Derby and Burton NHS Foundation Trust, reference number DHRD/2018/021.

This study was approved by the Health Research Authority, IRAS reference 247680.

No ethical approvals were required.

## Conflicts of interest

The project was funded through donation from a number of commercial organisations listed in the funding section of this manuscript.

Representatives from several organisations were present in their professional roles to offer their personal experiences, opinions and expertise during the refinement phase.

NUI kindly provided the NCCDs used in the study, on loan, free of charge.

The Professional Diving Academy, Dunoon, and Submarine Manufacturing and Products Limited, Preston, created the simulation complex for the refinement phase at no cost to the research budget.

None of the above individuals or organisations have had any control or influence over project design, delivery, data collection, data analysis, data interpretation or publication.

All research members and industry representatives participated either voluntarily, or as part of their normal employment; no attendees received extra payments for participation.

## CRediT authorship contribution statement

**Graham Johnson:** Writing – review & editing, Writing – original draft, Visualization, Validation, Resources, Project administration, Methodology, Investigation, Funding acquisition, Formal analysis, Data curation, Conceptualization. **Andrew Tabner:** Writing – review & editing, Writing – original draft, Visualization, Validation, Project administration, Methodology, Investigation, Funding acquisition, Formal analysis, Data curation, Conceptualization. **Nicholas Tilbury:** Writing – review & editing, Methodology, Investigation. **Alistair Wesson:** Writing – review & editing, Methodology, Investigation. **Gareth D. Hughes:** Writing – review & editing, Methodology, Investigation. **Rebecca Elder:** Writing – review & editing, Investigation. **Philip Bryson:** Writing – review & editing, Visualization, Resources, Methodology, Investigation, Funding acquisition, Conceptualization.

## Declaration of competing interest

The authors declare that they have no known competing financial interests or personal relationships that could have appeared to influence the work reported in this paper.
