# Supplementary material for: Development of an algorithm to guide management of cardiorespiratory arrest in a diving bell
Source: Resusc Plus. 2024 Jul 18;19:100724. doi: 10.1016/j.resplu.2024.100724 (PMC11295632; doi:10.1016/j.resplu.2024.100724)
Supplement: Supplementary Data 1 [file mmc1.docx]

**The Diver Resuscitation Group**

**Research/Medical Team**

Phil Bryson^1^ Diving and Hyperbaric Medicine Physician

Elisha Cousins^2^ Research Nurse

Rebecca Elder^3^ Specialty Trainee, Anaesthesia

Lianne Hufton^2^ Research Practitioner

Gareth D Hughes^2^ Emergency Medicine Charge Nurse

Gareth R Hughes^2^ Consultant Emergency Physician

Graham Johnson^2^ Consultant Emergency/Paediatric Emergency Medicine

David Jones^2^ Resuscitation and Clinical Skills Lead

Benjamin McGregor^4^ Offshore Medic/Critical Care Paramedic

Andrew Tabner^2^ Consultant Emergency Physician

Nicholas Tilbury^2^ Specialty Trainee Emergency Medicine/Pre-hospital Emergency Medicine

Alastair Wesson^4^ Dive Medical Technician

**Industry Team**

Keith Bailey^5^ Off-shore Medic

Simon Binstead^5^ Diving Operations Authority

Andrew Butler^6^ Diving Technical Authority & Lead

Mark Hamilton^7^ Diving Manager

David Hutchinson^8^ Retired - HM Inspector of Health and Safety

Ali Macleod^9^ Technical Advisor - Diving

Jerry Starling^10^ Direct of Diving and ROV Operations

Joni Watt^11^ Training Manager

Fredrik Bærheim^12^ HSEQ and Project Manager

**Diving Team**

Andrew Bell^7^ Saturation Diver

Vincent Bell^7^ Saturation Diver

Peter Billinghurst^7^ Saturation Diver

Don Campbell^7^  Saturation Diver

Adrian Corrigan^7^ Dive Supervisor

Gary Ferguson^7^ Saturation Diver

Dean Fox^7^ Saturation Diver

Paul Galloway^7^ Saturation Diver

Eamon Higgins^7^ Saturation Diver

Joe MccAfferty^7^ Saturation Diver

David Muir^7^ Saturation Diver

Ryan Shiach^7^ Saturation Diver

David Thompson^7^ Dive Supervisor

1. TAC Healthcare
2. University Hospitals of Derby and Burton NHS Foundation Trust
3. North West School of Anaesthesia, Manchester, UK
4. No Affiliation
5. Subsea 7
6. TechnipFMC
7. Boskalis
8. Well-Safe Solutions
9. International Marine Contractors Associations
10. RockSalt Subsea
11. Professional Diving Academy
12. NUI
